# A Dentist Abroad

**Published:** 1907-09

**Authors:** E. P. Beadles


### The American Journal of Dental Science

The Official Organ of the Florida, Georgia and South Carolina State Dental Societies

**Third Series**

VOL. XXXVIII SEPTEMBER, 1907 NO. 9

EDITORS

WM. GIRD BEECROFT, D. D. S., Madison, Wis. B. H. TEAGUE, D. D. S., Aiken, S. C.

Published on the 10th day of each month.
Subscription price $1.00 per year.
ENTERED AS SECOND-CLASS MATTER, MADISON, WIS.

Address all communications, both business and editorial, to
**The Dental Publishing Company, Madison, Wisconsin**

### A DENTIST ABROAD.

EDITOR JOURNAL:—I have recently had the pleasure of a short trip to Europe, a few observations may be of interest to your readers. The "Committee of Organization" knowing of my intention, gave me certain credentials, and extended through me an invitation to the profession in Europe to visit us in September and participate in the convention to be held at Norfolk, Virginia. All of your readers know of this meeting and none should fail to be present, a sight of the "big waters" will be well worth the trip.

The voyage from New York to Cherbourg, France, was exceptionally smooth, hence no sickness of the sea marred the pleasure of the passengers on the immense "Adriatic" of the White Star Line. A ship seven hundred and twenty-six feet long, the distance of more than fourteen city lots, fifty feet each, staggers the mind. This ship is twenty-five thousand tons burden, and carries a crew of sailors, stewards, waiters, officers, musicians, stokers, etc., of nearly one thousand. She is luxuriously fitted up with all the comforts of a great modern hotel. Swimming pool, cold baths, hot baths, turkish and electric baths, gymnasium, etc.

From Cherbourg, a ride by rail of six hours brings one to the great city of Paris. I was much interested in the old palaces, of Versaille and Fontainbleau situated a little way from the city itself. I visited Dr. Godon, well known in our profession, also the Davenport Brothers, American dentists, all treated me with consideration and expressed interest in our convention. There is an inconvenient custom among professional men in Paris, they have no signs upon their doors, not even a name. Of course I am speaking only of the ethical men. No man depreciates "display" more than myself, nor is anything more disgusting than a sign several feet in length; but for the convenience of all a name plate should be used. In Paris the city directory will give the name and number and perhaps the floor, but when you reach the place, which is usually in an arcade, there is absolutely no way to find the door of the office sought, unless happily one sees some occupant of the same building who may give the necessary information. The unethical go quite as far the other way. I saw one sign, on the top of a large building, the letters "American Dentists" extending over a space of at least forty feet in length. How one is shamed by all such! In addition to this the New York practice of having men on the street "handing out" advertising matter is much in vogue. Dentistry as a profession has a heavy load to carry in its upward march while it must drag in its wake men of this class.

I was impressed in Paris first with the sameness of the architecture. There seems to be a law which requires buildings to stop at a certain height, this makes all of them look very much alike. The windows are all made just as they were two hundred years ago—no improvements. Second, with the utter recklessness of drivers of all classes, I have heard that if a pedestrian is knocked down and run over, if he still lives, he is arrested instead of the driver, and fined for being in the way! Hence one must be quick, looking four ways at once. My third impression was with the great orderliness of the people. No drunkeness, no arrests did I see, though they are great wine drinkers. It was quite impossible to get a drink of water, and if wine is not ordered the price of it is invariably added to the bill. The fourth impression was the "open hand" not to give but to recieve. America is proving a gold mine to the people of Europe, and they are making the best of an opportunity. From Kings to peasants they will all take tips. It is reported that King Edward VII made two and one-half million dollars recently by one given him in regard to certain American railroad stocks.

There are some fine men in our profession in England as well as in France; but dental surgery as a whole must be at low ebb there. They "make teeth" in large numbers, but rarely do you see people in the street who show that the natural organs have had any attention from a dentist. The people have no appreciation of our skill and allow disease to bring them to the point of suffering, the dentist then advises extraction as the easiest way out of the difficulty, both for himself and for the patient. These causes produce a most deplorable condition as is easily seen, especially in the mouths of the English women.

I might discourse at length upon "curious customs" but will only mention one experience. Coming out of the British Museum one day, I desired to write a few lines to post and of course needed a sheet of paper, pen and ink. The Thackeray hotel is just opposite the entrance to the museum, walking in I asked an attendent for requisite material. He asked "are you a guest here?" "No, I am a stranger." "We can do nothing for you," was the curt reply, (though the serving ing classes in England are very polite.) This sounded strange to an American as all hotels in this country regard this courtesy as good advertising. One or two doors away was a sign, "Dr. Jones, Dentist." Surely a confrere will accommodate me with pen and paper. I confidently rang his bell and was ushered into a well furnished hall, containing works of art, statues and pictures, which much impressed me. The waiting room however was small and dark. In a few minutes a giant appeared in his shirt sleeves with my card in his hand. We addressed each other, I stated my business and explained that the matter pertained to our profession and that I wished to write a note to the president of the British Dental Association. This soiled specimen of huge flesh, with waxed mustache, looked me over in astonishment, showed me to the door with a waive of his hand, condescending no apology nor explanation except that dental associations were "not in his line." This was doubtless true, and I can vouch for it that politeness is not in his line. I sincerely hope that he is not an "American Dentist" in London, though his assumption of the title of "Doctor" would indicate that he is.

I suppose the English know their business best, but their law excluding American dentists who are gentlemen and accomplished members of their profession is not for the benefit of the English dentists nor the English people. But my notes are too lengthy.

Very truly yours

E. P. Beadles, D. D. S.

